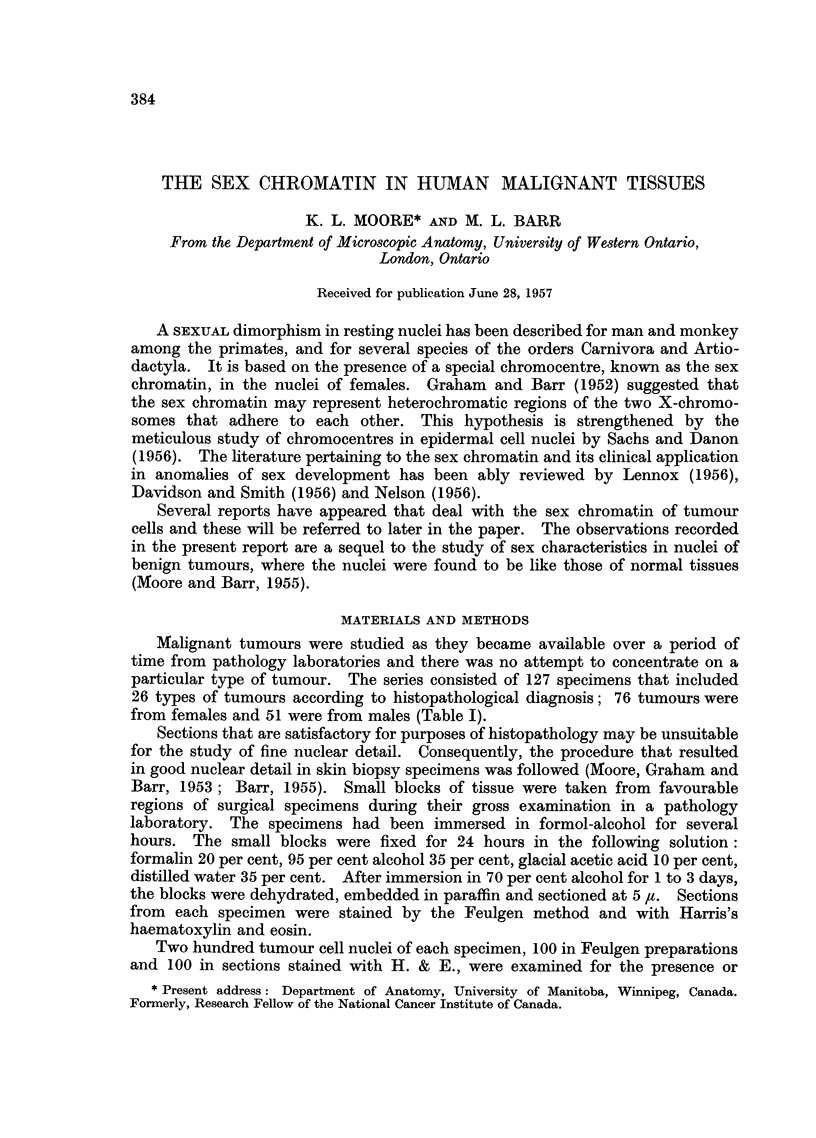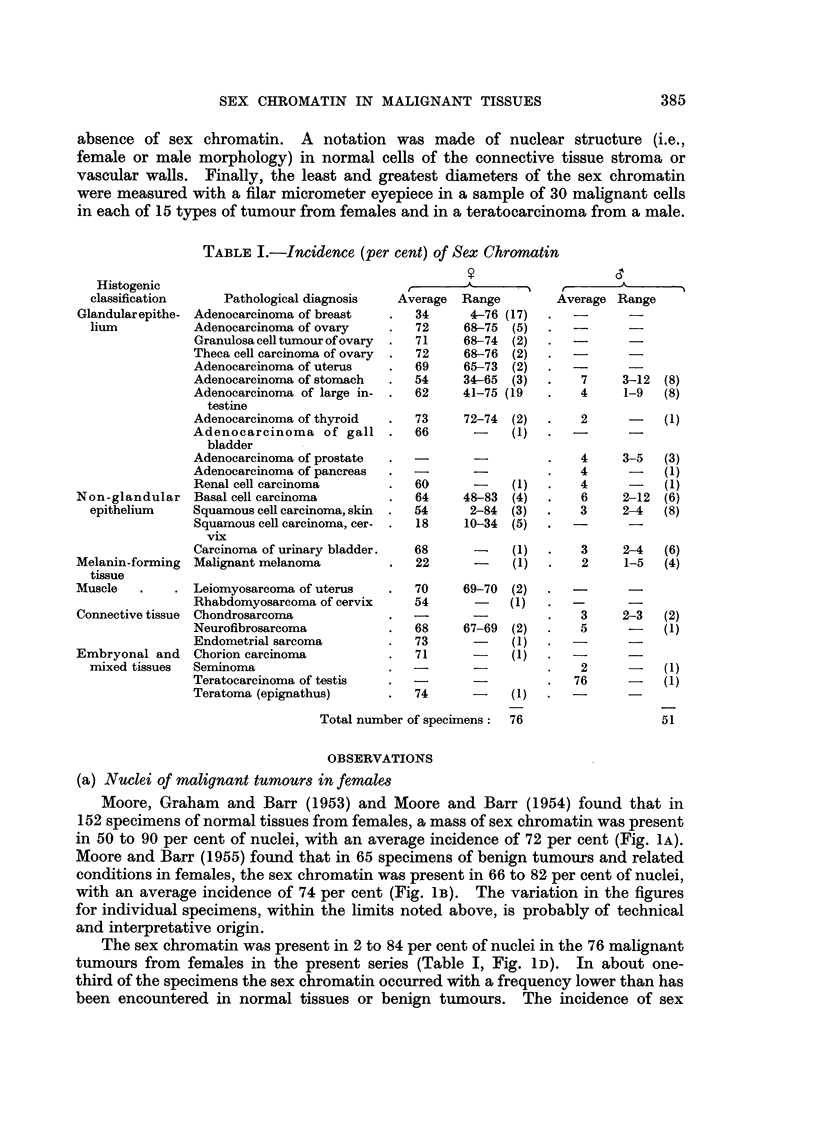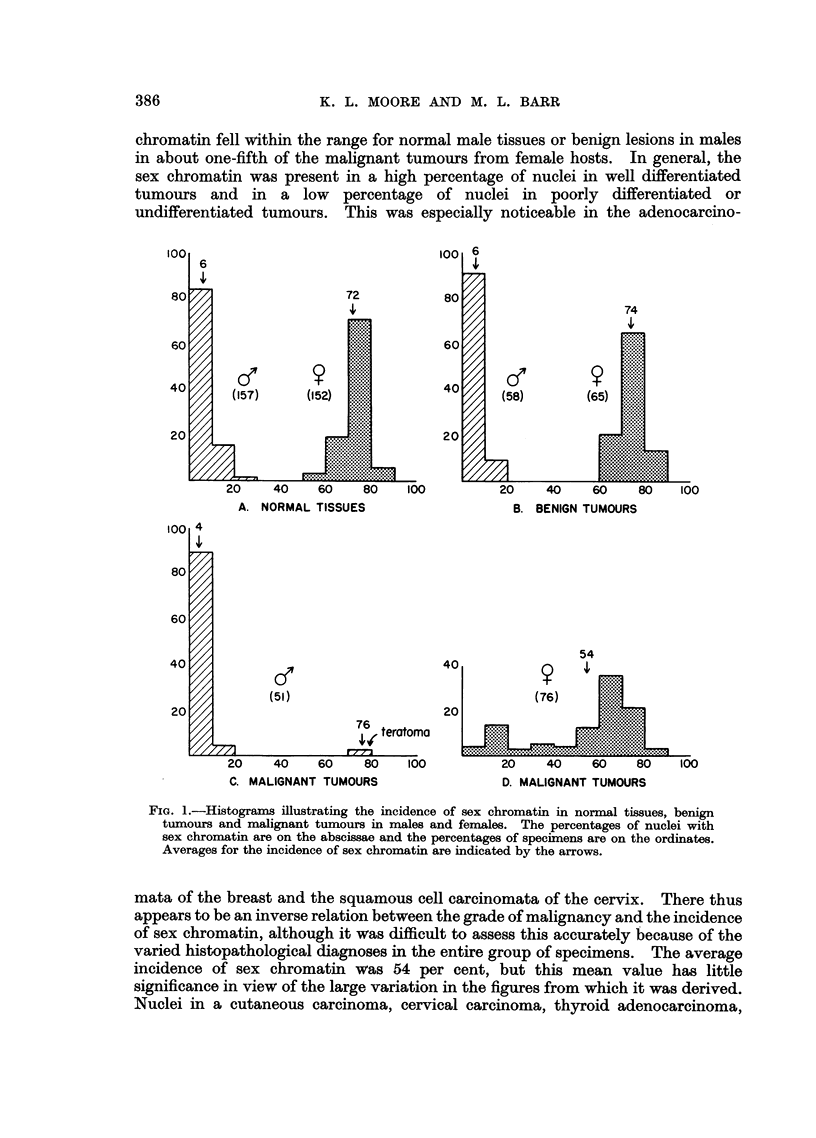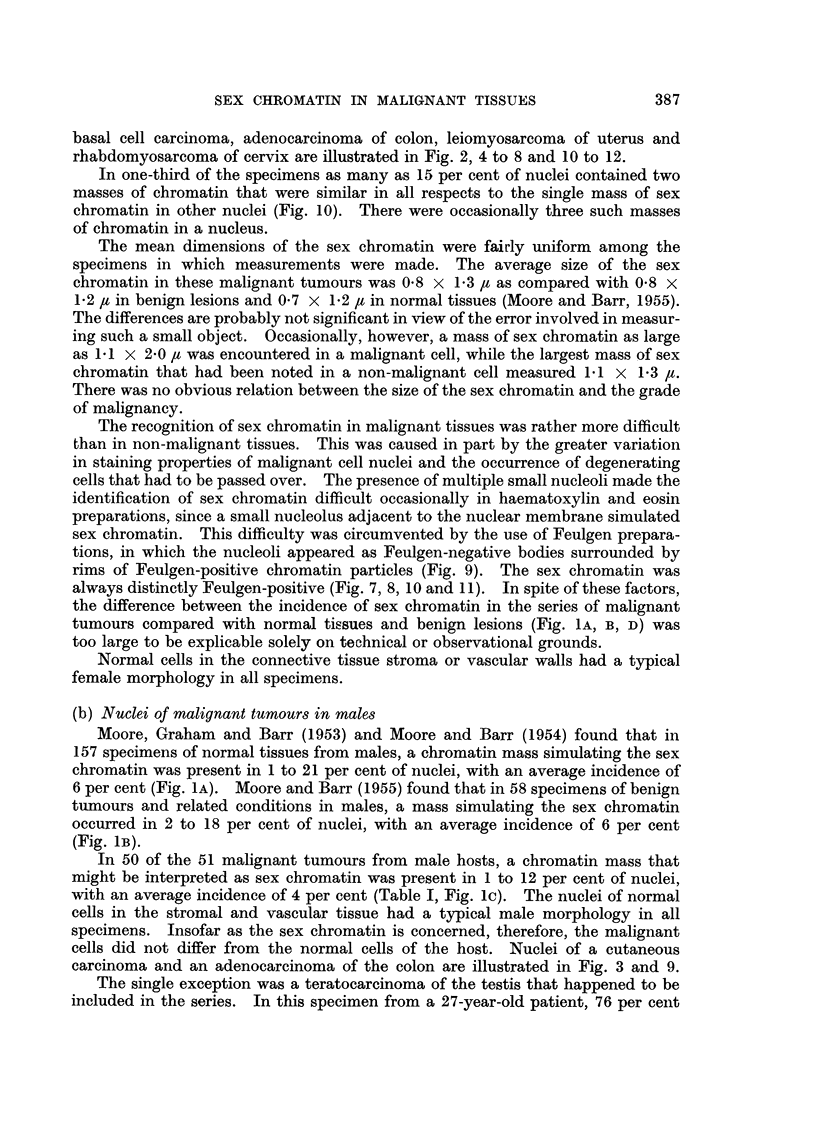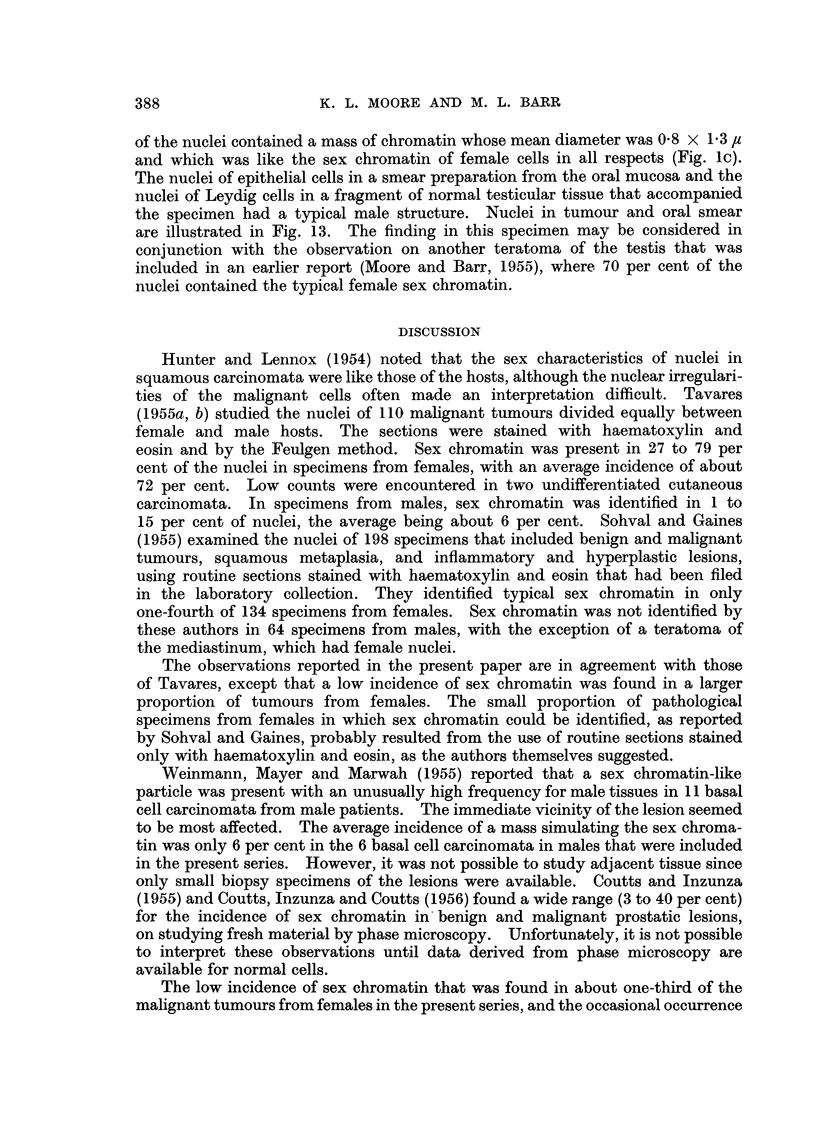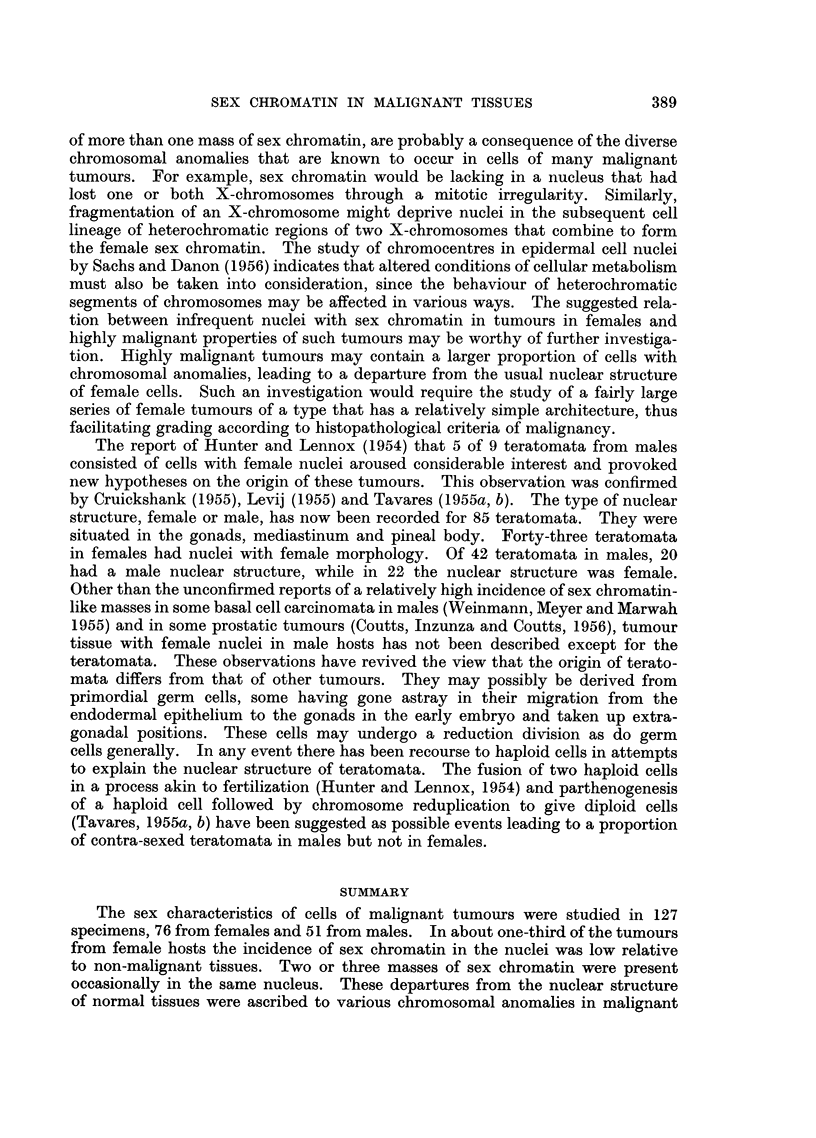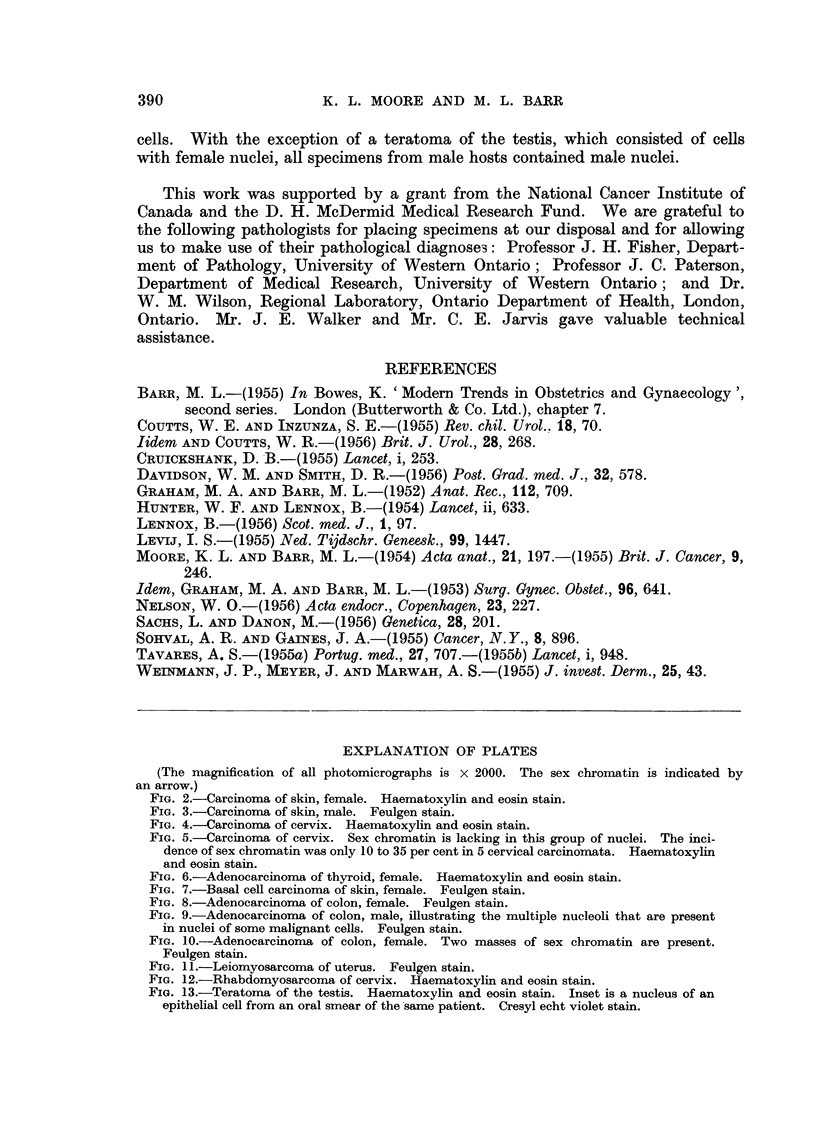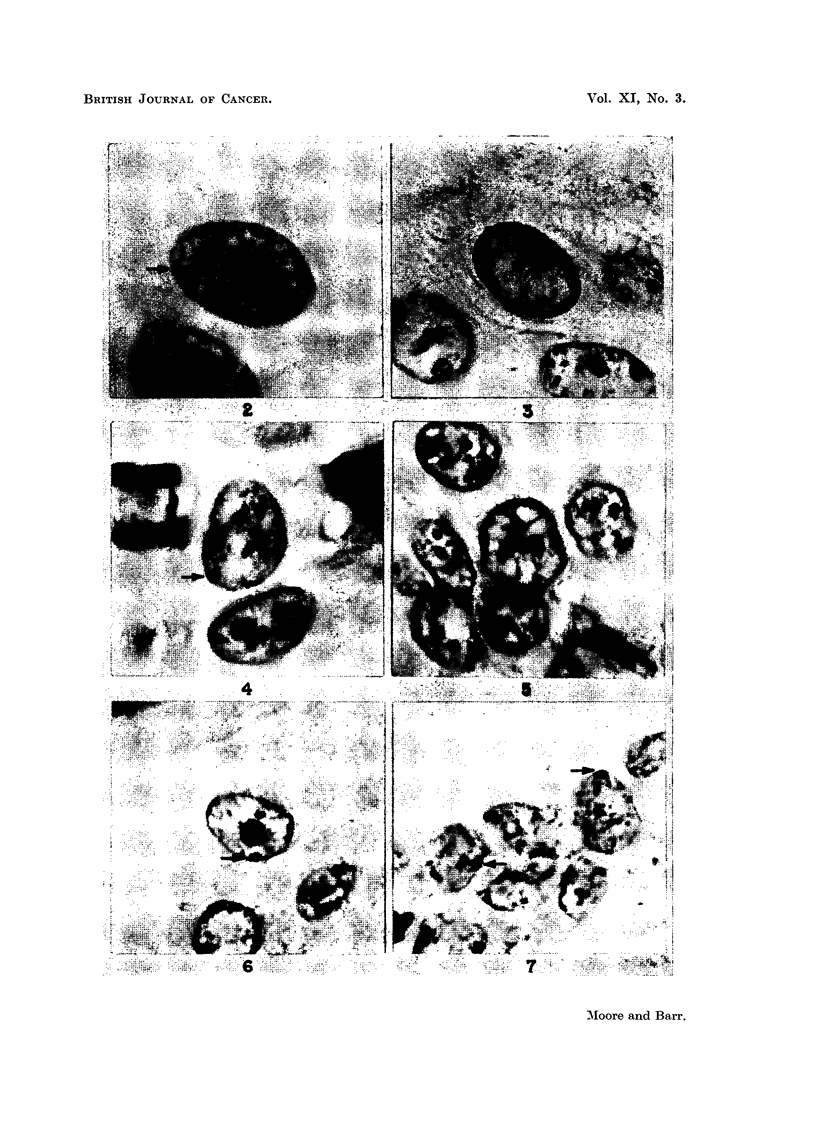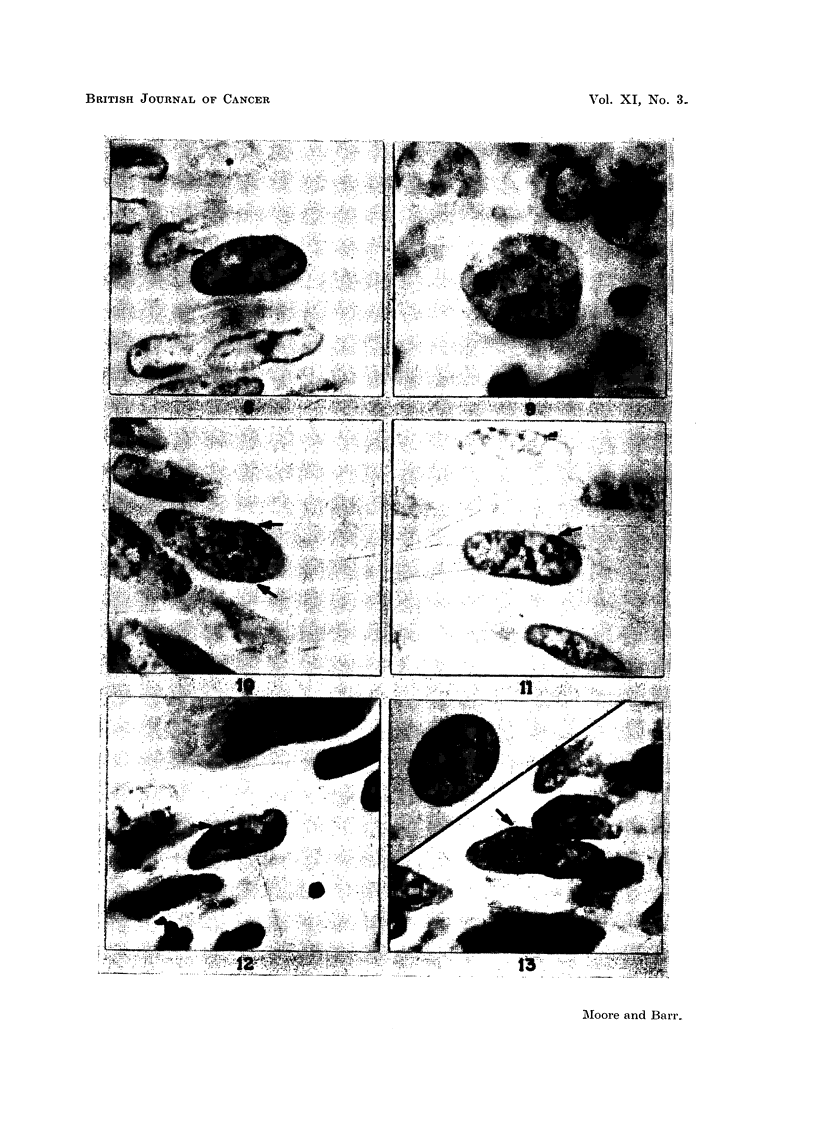# The Sex Chromatin in Human Malignant Tissues

**DOI:** 10.1038/bjc.1957.45

**Published:** 1957-09

**Authors:** K. L. Moore, M. L. Barr

## Abstract

**Images:**


### THE SEX CHROMATIN IN HUMAN MALIGNANT TISSUES

K. L. MOORE* AND M. L. BARR

*From the Department of Microscopic Anatomy, University of Western Ontario, London, Ontario*

Received for publication June 28, 1957

A SEXUAL dimorphism in resting nuclei has been described for man and monkey among the primates, and for several species of the orders Carnivora and Artiodactyla. It is based on the presence of a special chromocentre, known as the sex chromatin, in the nuclei of females. Graham and Barr (1952) suggested that the sex chromatin may represent heterochromatic regions of the two X-chromosomes that adhere to each other. This hypothesis is strengthened by the meticulous study of chromocentres in epidermal cell nuclei by Sachs and Danon (1956). The literature pertaining to the sex chromatin and its clinical application in anomalies of sex development has been ably reviewed by Lennox (1956), Davidson and Smith (1956) and Nelson (1956).

Several reports have appeared that deal with the sex chromatin of tumour cells and these will be referred to later in the paper. The observations recorded in the present report are a sequel to the study of sex characteristics in nuclei of benign tumours, where the nuclei were found to be like those of normal tissues (Moore and Barr, 1955).

### MATERIALS AND METHODS

Malignant tumours were studied as they became available over a period of time from pathology laboratories and there was no attempt to concentrate on a particular type of tumour. The series consisted of 127 specimens that included 26 types of tumours according to histopathological diagnosis; 76 tumours were from females and 51 were from males (Table I).

Sections that are satisfactory for purposes of histopathology may be unsuitable for the study of fine nuclear detail. Consequently, the procedure that resulted in good nuclear detail in skin biopsy specimens was followed (Moore, Graham and Barr, 1953; Barr, 1955). Small blocks of tissue were taken from favourable regions of surgical specimens during their gross examination in a pathology laboratory. The specimens had been immersed in formol-alcohol for several hours. The small blocks were fixed for 24 hours in the following solution: formalin 20 per cent, 95 per cent alcohol 35 per cent, glacial acetic acid 10 per cent, distilled water 35 per cent. After immersion in 70 per cent alcohol for 1 to 3 days, the blocks were dehydrated, embedded in paraffin and sectioned at 5 $\mu$. Sections from each specimen were stained by the Feulgen method and with Harris's haematoxylin and eosin.

Two hundred tumour cell nuclei of each specimen, 100 in Feulgen preparations and 100 in sections stained with H. & E., were examined for the presence or

* Present address: Department of Anatomy, University of Manitoba, Winnipeg, Canada. Formerly, Research Fellow of the National Cancer Institute of Canada.

absence of sex chromatin. A notation was made of nuclear structure (i.e., female or male morphology) in normal cells of the connective tissue stroma or vascular walls. Finally, the least and greatest diameters of the sex chromatin were measured with a filar micrometer eyepiece in a sample of 30 malignant cells in each of 15 types of tumour from females and in a teratocarcinoma from a male.

TABLE I.—*Incidence (per cent) of Sex Chromatin*

| Histogenic classification | Pathological diagnosis | ♀ Average | ♀ Range | | ♂ Average | ♂ Range | |
|---|---|---|---|---|---|---|---|
| Glandular epithelium | Adenocarcinoma of breast | 34 | 4–76 | (17) | — | — | |
| | Adenocarcinoma of ovary | 72 | 68–75 | (5) | — | — | |
| | Granulosa cell tumour of ovary | 71 | 68–74 | (2) | — | — | |
| | Theca cell carcinoma of ovary | 72 | 68–76 | (2) | — | — | |
| | Adenocarcinoma of uterus | 69 | 65–73 | (2) | — | — | |
| | Adenocarcinoma of stomach | 54 | 34–65 | (3) | 7 | 3–12 | (8) |
| | Adenocarcinoma of large intestine | 62 | 41–75 | (19 | 4 | 1–9 | (8) |
| | Adenocarcinoma of thyroid | 73 | 72–74 | (2) | 2 | — | (1) |
| | Adenocarcinoma of gall bladder | 66 | — | (1) | — | — | |
| | Adenocarcinoma of prostate | — | — | | 4 | 3–5 | (3) |
| | Adenocarcinoma of pancreas | — | — | | 4 | — | (1) |
| | Renal cell carcinoma | 60 | — | (1) | 4 | — | (1) |
| Non-glandular epithelium | Basal cell carcinoma | 64 | 48–83 | (4) | 6 | 2–12 | (6) |
| | Squamous cell carcinoma, skin | 54 | 2–84 | (3) | 3 | 2–4 | (8) |
| | Squamous cell carcinoma, cervix | 18 | 10–34 | (5) | — | — | |
| | Carcinoma of urinary bladder | 68 | — | (1) | 3 | 2–4 | (6) |
| Melanin-forming tissue | Malignant melanoma | 22 | — | (1) | 2 | 1–5 | (4) |
| Muscle | Leiomyosarcoma of uterus | 70 | 69–70 | (2) | — | — | |
| | Rhabdomyosarcoma of cervix | 54 | — | (1) | — | — | |
| Connective tissue | Chondrosarcoma | — | — | | 3 | 2–3 | (2) |
| | Neurofibrosarcoma | 68 | 67–69 | (2) | 5 | — | (1) |
| | Endometrial sarcoma | 73 | — | (1) | — | — | |
| Embryonal and mixed tissues | Chorion carcinoma | 71 | — | (1) | — | — | |
| | Seminoma | — | — | | 2 | — | (1) |
| | Teratocarcinoma of testis | — | — | | 76 | — | (1) |
| | Teratoma (epignathus) | 74 | — | (1) | — | — | |
| | Total number of specimens : | | | 76 | | | 51 |

OBSERVATIONS

(a) *Nuclei of malignant tumours in females*

Moore, Graham and Barr (1953) and Moore and Barr (1954) found that in 152 specimens of normal tissues from females, a mass of sex chromatin was present in 50 to 90 per cent of nuclei, with an average incidence of 72 per cent (Fig. 1A). Moore and Barr (1955) found that in 65 specimens of benign tumours and related conditions in females, the sex chromatin was present in 66 to 82 per cent of nuclei, with an average incidence of 74 per cent (Fig. 1B). The variation in the figures for individual specimens, within the limits noted above, is probably of technical and interpretative origin.

The sex chromatin was present in 2 to 84 per cent of nuclei in the 76 malignant tumours from females in the present series (Table I, Fig. 1D). In about one-third of the specimens the sex chromatin occurred with a frequency lower than has been encountered in normal tissues or benign tumours. The incidence of sex chromatin fell within the range for normal male tissues or benign lesions in males in about one-fifth of the malignant tumours from female hosts. In general, the sex chromatin was present in a high percentage of nuclei in well differentiated tumours and in a low percentage of nuclei in poorly differentiated or undifferentiated tumours. This was especially noticeable in the adenocarcino-

FIG. 1.—Histograms illustrating the incidence of sex chromatin in normal tissues, benign tumours and malignant tumours in males and females. The percentages of nuclei with sex chromatin are on the abscissae and the percentages of specimens are on the ordinates. Averages for the incidence of sex chromatin are indicated by the arrows.

mata of the breast and the squamous cell carcinomata of the cervix. There thus appears to be an inverse relation between the grade of malignancy and the incidence of sex chromatin, although it was difficult to assess this accurately because of the varied histopathological diagnoses in the entire group of specimens. The average incidence of sex chromatin was 54 per cent, but this mean value has little significance in view of the large variation in the figures from which it was derived. Nuclei in a cutaneous carcinoma, cervical carcinoma, thyroid adenocarcinoma, basal cell carcinoma, adenocarcinoma of colon, leiomyosarcoma of uterus and rhabdomyosarcoma of cervix are illustrated in Fig. 2, 4 to 8 and 10 to 12.

In one-third of the specimens as many as 15 per cent of nuclei contained two masses of chromatin that were similar in all respects to the single mass of sex chromatin in other nuclei (Fig. 10). There were occasionally three such masses of chromatin in a nucleus.

The mean dimensions of the sex chromatin were fairly uniform among the specimens in which measurements were made. The average size of the sex chromatin in these malignant tumours was $0{\cdot}8 \times 1{\cdot}3\ \mu$ as compared with $0{\cdot}8 \times 1{\cdot}2\ \mu$ in benign lesions and $0{\cdot}7 \times 1{\cdot}2\ \mu$ in normal tissues (Moore and Barr, 1955). The differences are probably not significant in view of the error involved in measuring such a small object. Occasionally, however, a mass of sex chromatin as large as $1{\cdot}1 \times 2{\cdot}0\ \mu$ was encountered in a malignant cell, while the largest mass of sex chromatin that had been noted in a non-malignant cell measured $1{\cdot}1 \times 1{\cdot}3\ \mu$. There was no obvious relation between the size of the sex chromatin and the grade of malignancy.

The recognition of sex chromatin in malignant tissues was rather more difficult than in non-malignant tissues. This was caused in part by the greater variation in staining properties of malignant cell nuclei and the occurrence of degenerating cells that had to be passed over. The presence of multiple small nucleoli made the identification of sex chromatin difficult occasionally in haematoxylin and eosin preparations, since a small nucleolus adjacent to the nuclear membrane simulated sex chromatin. This difficulty was circumvented by the use of Feulgen preparations, in which the nucleoli appeared as Feulgen-negative bodies surrounded by rims of Feulgen-positive chromatin particles (Fig. 9). The sex chromatin was always distinctly Feulgen-positive (Fig. 7, 8, 10 and 11). In spite of these factors, the difference between the incidence of sex chromatin in the series of malignant tumours compared with normal tissues and benign lesions (Fig. 1A, B, D) was too large to be explicable solely on technical or observational grounds.

Normal cells in the connective tissue stroma or vascular walls had a typical female morphology in all specimens.

(b) *Nuclei of malignant tumours in males*

Moore, Graham and Barr (1953) and Moore and Barr (1954) found that in 157 specimens of normal tissues from males, a chromatin mass simulating the sex chromatin was present in 1 to 21 per cent of nuclei, with an average incidence of 6 per cent (Fig. 1A). Moore and Barr (1955) found that in 58 specimens of benign tumours and related conditions in males, a mass simulating the sex chromatin occurred in 2 to 18 per cent of nuclei, with an average incidence of 6 per cent (Fig. 1B).

In 50 of the 51 malignant tumours from male hosts, a chromatin mass that might be interpreted as sex chromatin was present in 1 to 12 per cent of nuclei, with an average incidence of 4 per cent (Table I, Fig. 1C). The nuclei of normal cells in the stromal and vascular tissue had a typical male morphology in all specimens. Insofar as the sex chromatin is concerned, therefore, the malignant cells did not differ from the normal cells of the host. Nuclei of a cutaneous carcinoma and an adenocarcinoma of the colon are illustrated in Fig. 3 and 9.

The single exception was a teratocarcinoma of the testis that happened to be included in the series. In this specimen from a 27-year-old patient, 76 per cent of the nuclei contained a mass of chromatin whose mean diameter was $0 \cdot 8 \times 1 \cdot 3\ \mu$ and which was like the sex chromatin of female cells in all respects (Fig. 1c). The nuclei of epithelial cells in a smear preparation from the oral mucosa and the nuclei of Leydig cells in a fragment of normal testicular tissue that accompanied the specimen had a typical male structure. Nuclei in tumour and oral smear are illustrated in Fig. 13. The finding in this specimen may be considered in conjunction with the observation on another teratoma of the testis that was included in an earlier report (Moore and Barr, 1955), where 70 per cent of the nuclei contained the typical female sex chromatin.

### DISCUSSION

Hunter and Lennox (1954) noted that the sex characteristics of nuclei in squamous carcinomata were like those of the hosts, although the nuclear irregularities of the malignant cells often made an interpretation difficult. Tavares (1955*a*, *b*) studied the nuclei of 110 malignant tumours divided equally between female and male hosts. The sections were stained with haematoxylin and eosin and by the Feulgen method. Sex chromatin was present in 27 to 79 per cent of the nuclei in specimens from females, with an average incidence of about 72 per cent. Low counts were encountered in two undifferentiated cutaneous carcinomata. In specimens from males, sex chromatin was identified in 1 to 15 per cent of nuclei, the average being about 6 per cent. Sohval and Gaines (1955) examined the nuclei of 198 specimens that included benign and malignant tumours, squamous metaplasia, and inflammatory and hyperplastic lesions, using routine sections stained with haematoxylin and eosin that had been filed in the laboratory collection. They identified typical sex chromatin in only one-fourth of 134 specimens from females. Sex chromatin was not identified by these authors in 64 specimens from males, with the exception of a teratoma of the mediastinum, which had female nuclei.

The observations reported in the present paper are in agreement with those of Tavares, except that a low incidence of sex chromatin was found in a larger proportion of tumours from females. The small proportion of pathological specimens from females in which sex chromatin could be identified, as reported by Sohval and Gaines, probably resulted from the use of routine sections stained only with haematoxylin and eosin, as the authors themselves suggested.

Weinmann, Mayer and Marwah (1955) reported that a sex chromatin-like particle was present with an unusually high frequency for male tissues in 11 basal cell carcinomata from male patients. The immediate vicinity of the lesion seemed to be most affected. The average incidence of a mass simulating the sex chromatin was only 6 per cent in the 6 basal cell carcinomata in males that were included in the present series. However, it was not possible to study adjacent tissue since only small biopsy specimens of the lesions were available. Coutts and Inzunza (1955) and Coutts, Inzunza and Coutts (1956) found a wide range (3 to 40 per cent) for the incidence of sex chromatin in benign and malignant prostatic lesions, on studying fresh material by phase microscopy. Unfortunately, it is not possible to interpret these observations until data derived from phase microscopy are available for normal cells.

The low incidence of sex chromatin that was found in about one-third of the malignant tumours from females in the present series, and the occasional occurrence of more than one mass of sex chromatin, are probably a consequence of the diverse chromosomal anomalies that are known to occur in cells of many malignant tumours. For example, sex chromatin would be lacking in a nucleus that had lost one or both X-chromosomes through a mitotic irregularity. Similarly, fragmentation of an X-chromosome might deprive nuclei in the subsequent cell lineage of heterochromatic regions of two X-chromosomes that combine to form the female sex chromatin. The study of chromocentres in epidermal cell nuclei by Sachs and Danon (1956) indicates that altered conditions of cellular metabolism must also be taken into consideration, since the behaviour of heterochromatic segments of chromosomes may be affected in various ways. The suggested relation between infrequent nuclei with sex chromatin in tumours in females and highly malignant properties of such tumours may be worthy of further investigation. Highly malignant tumours may contain a larger proportion of cells with chromosomal anomalies, leading to a departure from the usual nuclear structure of female cells. Such an investigation would require the study of a fairly large series of female tumours of a type that has a relatively simple architecture, thus facilitating grading according to histopathological criteria of malignancy.

The report of Hunter and Lennox (1954) that 5 of 9 teratomata from males consisted of cells with female nuclei aroused considerable interest and provoked new hypotheses on the origin of these tumours. This observation was confirmed by Cruickshank (1955), Levij (1955) and Tavares (1955*a*, *b*). The type of nuclear structure, female or male, has now been recorded for 85 teratomata. They were situated in the gonads, mediastinum and pineal body. Forty-three teratomata in females had nuclei with female morphology. Of 42 teratomata in males, 20 had a male nuclear structure, while in 22 the nuclear structure was female. Other than the unconfirmed reports of a relatively high incidence of sex chromatin-like masses in some basal cell carcinomata in males (Weinmann, Meyer and Marwah 1955) and in some prostatic tumours (Coutts, Inzunza and Coutts, 1956), tumour tissue with female nuclei in male hosts has not been described except for the teratomata. These observations have revived the view that the origin of teratomata differs from that of other tumours. They may possibly be derived from primordial germ cells, some having gone astray in their migration from the endodermal epithelium to the gonads in the early embryo and taken up extragonadal positions. These cells may undergo a reduction division as do germ cells generally. In any event there has been recourse to haploid cells in attempts to explain the nuclear structure of teratomata. The fusion of two haploid cells in a process akin to fertilization (Hunter and Lennox, 1954) and parthenogenesis of a haploid cell followed by chromosome reduplication to give diploid cells (Tavares, 1955*a*, *b*) have been suggested as possible events leading to a proportion of contra-sexed teratomata in males but not in females.

### SUMMARY

The sex characteristics of cells of malignant tumours were studied in 127 specimens, 76 from females and 51 from males. In about one-third of the tumours from female hosts the incidence of sex chromatin in the nuclei was low relative to non-malignant tissues. Two or three masses of sex chromatin were present occasionally in the same nucleus. These departures from the nuclear structure of normal tissues were ascribed to various chromosomal anomalies in malignant cells. With the exception of a teratoma of the testis, which consisted of cells with female nuclei, all specimens from male hosts contained male nuclei.

This work was supported by a grant from the National Cancer Institute of Canada and the D. H. McDermid Medical Research Fund. We are grateful to the following pathologists for placing specimens at our disposal and for allowing us to make use of their pathological diagnoses: Professor J. H. Fisher, Department of Pathology, University of Western Ontario; Professor J. C. Paterson, Department of Medical Research, University of Western Ontario; and Dr. W. M. Wilson, Regional Laboratory, Ontario Department of Health, London, Ontario. Mr. J. E. Walker and Mr. C. E. Jarvis gave valuable technical assistance.

### REFERENCES

BARR, M. L.—(1955) *In* Bowes, K. 'Modern Trends in Obstetrics and Gynaecology', second series. London (Butterworth & Co. Ltd.), chapter 7.
COUTTS, W. E. AND INZUNZA, S. E.—(1955) *Rev. chil. Urol.*, **18**, 70.
*Iidem* AND COUTTS, W. R.—(1956) *Brit. J. Urol.*, **28**, 268.
CRUICKSHANK, D. B.—(1955) *Lancet*, i, 253.
DAVIDSON, W. M. AND SMITH, D. R.—(1956) *Post. Grad. med. J.*, **32**, 578.
GRAHAM, M. A. AND BARR, M. L.—(1952) *Anat. Rec.*, **112**, 709.
HUNTER, W. F. AND LENNOX, B.—(1954) *Lancet*, ii, 633.
LENNOX, B.—(1956) *Scot. med. J.*, **1**, 97.
LEVIJ, I. S.—(1955) *Ned. Tijdschr. Geneesk.*, **99**, 1447.
MOORE, K. L. AND BARR, M. L.—(1954) *Acta anat.*, **21**, 197.—(1955) *Brit. J. Cancer*, **9**, 246.
*Idem*, GRAHAM, M. A. AND BARR, M. L.—(1953) *Surg. Gynec. Obstet.*, **96**, 641.
NELSON, W. O.—(1956) *Acta endocr., Copenhagen*, **23**, 227.
SACHS, L. AND DANON, M.—(1956) *Genetica*, **28**, 201.
SOHVAL, A. R. AND GAINES, J. A.—(1955) *Cancer, N.Y.*, **8**, 896.
TAVARES, A. S.—(1955*a*) *Portug. med.*, **27**, 707.—(1955*b*) *Lancet*, i, 948.
WEINMANN, J. P., MEYER, J. AND MARWAH, A. S.—(1955) *J. invest. Derm.*, **25**, 43.

---

### EXPLANATION OF PLATES

(The magnification of all photomicrographs is × 2000. The sex chromatin is indicated by an arrow.)

FIG. 2.—Carcinoma of skin, female. Haematoxylin and eosin stain.
FIG. 3.—Carcinoma of skin, male. Feulgen stain.
FIG. 4.—Carcinoma of cervix. Haematoxylin and eosin stain.
FIG. 5.—Carcinoma of cervix. Sex chromatin is lacking in this group of nuclei. The incidence of sex chromatin was only 10 to 35 per cent in 5 cervical carcinomata. Haematoxylin and eosin stain.
FIG. 6.—Adenocarcinoma of thyroid, female. Haematoxylin and eosin stain.
FIG. 7.—Basal cell carcinoma of skin, female. Feulgen stain.
FIG. 8.—Adenocarcinoma of colon, female. Feulgen stain.
FIG. 9.—Adenocarcinoma of colon, male, illustrating the multiple nucleoli that are present in nuclei of some malignant cells. Feulgen stain.
FIG. 10.—Adenocarcinoma of colon, female. Two masses of sex chromatin are present. Feulgen stain.
FIG. 11.—Leiomyosarcoma of uterus. Feulgen stain.
FIG. 12.—Rhabdomyosarcoma of cervix. Haematoxylin and eosin stain.
FIG. 13.—Teratoma of the testis. Haematoxylin and eosin stain. Inset is a nucleus of an epithelial cell from an oral smear of the same patient. Cresyl echt violet stain.

Moore and Barr.